# Acute Myeloid Leukemia in Children: Emerging Paradigms in Genetics and New Approaches to Therapy

**DOI:** 10.1007/s11912-020-01009-3

**Published:** 2021-01-13

**Authors:** Shannon E. Conneely, Alexandra M. Stevens

**Affiliations:** Department of Pediatric Hematology/Oncology, Baylor College of Medicine/Texas Children’s Hospital, 6701 Fannin, Suite 1510, Houston, TX 77030 USA

**Keywords:** Risk stratification, Epigenetic, Chemotherapy, Immunotherapy, Tyrosine kinase, Outcomes

## Abstract

**Purpose of Review:**

Acute myeloid leukemia (AML) in children remains a challenging disease to cure with suboptimal outcomes particularly when compared to the more common lymphoid leukemias. Recent advances in the genetic characterization of AML have enhanced understanding of individualized patient risk, which has also led to the development of new therapeutic strategies. Here, we review key cytogenetic and molecular features of pediatric AML and how new therapies are being used to improve outcomes.

**Recent Findings:**

Recent studies have revealed an increasing number of mutations, including *WT1*, *CBFA2T3*-*GLIS2*, and *KAT6A* fusions, *DEK*-*NUP214* and *NUP98* fusions, and specific *KMT2A* rearrangements, which are associated with poor outcomes. However, outcomes are starting to improve with the addition of therapies such as gemtuzumab ozogamicin and *FLT3* inhibitors, initially developed in adult AML.

**Summary:**

The combination of advanced risk stratification and ongoing improvements and innovations in treatment strategy will undoubtedly lead to better outcomes for children with AML.

## Introduction

Pediatric acute myeloid leukemia (AML) is a heterogeneous disease with generally poor outcomes compared to childhood lymphoid leukemia. Decades of coordinated efforts through cooperative group trials have improved our understanding of the unique biology underlying pediatric AML and patient outcomes. However, relapse remains frequent with limited available treatment options. As AML is more common and heavily researched in older adults, the field of pediatric AML has advanced in the understanding of AML biology and novel therapeutic strategies by translation of adult studies into the realm of pediatrics. However, there are substantial differences in the mutational landscape in pediatric versus adult AML and tolerance to treatment regimens, thus requiring dedicated risk stratification and treatment considerations in children.

Compared to adults, children with AML have superior outcomes due to fewer adverse genetic mutations and the ability to tolerate the high-intensity chemotherapy currently necessary for cure. While complete remission (CR) rates are high in pediatric AML at approximately 90%, event-free survival (EFS) and overall survival (OS) remain suboptimal at 45% and 65%, respectively, at 3 years, and nearly half of children will relapse [1, 2]. Even in the low-risk genetic groups, relapse remains common at up to 35%. Unfortunately, children at the highest risk of relapse related to poor genetic features have dismal outcomes altogether and continue to require stem cell transplant (SCT) to achieve cure, with only one in three surviving at 3 years [1].

Treatment decisions, namely the need for SCT, in pediatric AML are primarily driven by genetic risk classification which is quickly evolving. The World Health Organization (WHO) began defining recurrent genetic groups in myeloid malignancies in 2001 due to high incidence of specific mutations in AML, unique underlying biology, and comparable outcomes amongst patients with these lesions [3]. However, these definitions are primarily based on adult studies, and while an overlap between genetic features in adults and children exists, assigning risk categories in children requires special consideration. Currently, t(15;17), the defining genetic rearrangement of acute promyelocytic leukemia, is excluded from most AML studies due to highly specific therapy and superior outcomes and will not be discussed here. Favorable-risk genetics such as inv(16), t(8;21), and *CEBPA* and *NPM1* mutations may be cured with chemotherapy alone [4–6]. In contrast, monosomy 7 and monosomy 5 or 5q deletions are high-risk (HR) features which require SCT in first remission for the best outcomes [7, 8]. Rearrangements of *KMT2A*, located on chromosome 11q23, were also included in initial WHO classifications, but the prognostic significance was unclear until recently [9, 10••]. As advanced testing abilities and mutation profiling have become more readily available, these genetic risk groups are rapidly changing. Through collaborative efforts such as the Therapeutically Applicable Research to Generate Effective Treatments (TARGET) AML initiative, in-depth descriptions of pediatric AML mutations and their effects on outcomes have been achieved [10••].

Along with improving our understanding of the biology of AML, identification of recurrent mutations has provided an opportunity to develop new treatment approaches. Targeted therapeutics, epigenetic modifiers, immune-based strategies, and novel metabolic pathway inhibition have all been utilized recently with varying degrees of success in adult AML. These strategies are now being employed in children with the aim of improving cure rates and providing more options in the relapse setting. Here, we discuss the current progress in understanding the unique mutational landscape of pediatric AML, implications on prognosis, and the use of new treatment strategies to optimize therapy and outcomes in children with AML.

## Genetics

### Established Genetic Risk Factors

Risk stratification in pediatric AML has previously been based on the presence of a handful of genetic features and responses to therapy. Those with favorable genetics include t(8;21), inv(16), *NPM1* mutations, and *CEBPA* mutations, each of which carries EFS and OS rates of approximately 65–70% and 80%, respectively [1, 4, 11]. t(8;21) and inv(16), collectively termed core binding factor (CBF) AML, represent the most common cytogenetic subgroup in pediatric AML accounting for 20–25% of cases [1, 10••, 11]. Mutations in *NPM1* and *CEBPA* are more recent additions to the favorable prognostic group and are similarly associated with an improved prognosis in both children and adults [5]. *CEBPA* encodes a transcription factor that regulates expression of myeloid-specific genes with recurrent mutations occurring in two functional domains in approximately 4% of pediatric AML [4]. *NPM1* is a primarily nucleolar protein which plays a role in regulating p53 function and other cellular processes and, when mutated in AML, aberrantly localizes to the cytoplasm [5]. *NPM1* mutations are more common with increasing age but do occur in about 5–10% of children compared to 30% of adults [5, 10••, 12, 13•]. Pediatric studies demonstrate a higher EFS and OS with *NPM1* mutations, particularly in cytogenetically normal AML or when they occur in the context of HR mutations where the presence of mutated *NPM1* may improve outcomes compared to the HR genetic feature alone [5, 6, 10••].

In contrast to the favorable outcomes associated with CBF AML and *NPM1* or *CEBPA* mutations, certain HR features have consistently shown poor outcomes in children and adults over the decades. This includes monosomy of chromosome 7 or 5 or deletion of 5q. These HR features are more common in adults and collectively occur in less than 5% of pediatric AML patients [14]. Of these, monosomy 7 is more common and carries a high rate of induction failure, EFS of 17–29%, and OS of 32%, a significantly worse prognosis than the HR group as a whole (EFS of 28%; OS of 48%) [1, 14–17]. The list of mutations conferring an HR status and overall poor outcome continues to expand as research reveals new mutations and prognostic relevance. Identification of such cytogenetic and molecular changes is the first step to targeting dependencies for these HR subtypes and ultimately improving outcomes.

### Tyrosine Kinase Mutations

#### FLT3

FMS-like tyrosine kinase 3 (*FLT3*) is a recurrently mutated gene in AML, occurring in 20–25% of children and 30% of adults [10••, 13•, 18]. *FLT3* mutations lead to constitutive activation of the tyrosine kinase domain (TKD) causing uninhibited growth and are characterized as either TKD or internal tandem duplication (ITD) mutations with *FLT3*-ITD being significantly more common in children (Table 1) [13•]. The prognostic relevance of the *FLT3-*ITD allelic ratio (AR) has been refined over the last decade. Previous trials demonstrated that an AR > 0.4 conferred a poorer prognosis compared to an AR < 0.4, and so *FLT3-*targeted therapy was included only for the high AR group [19]. However, updated analysis has suggested that an AR > 0.1 is sufficient to confer unfavorable EFS of 25–30% and OS of 60% [20•]. Therefore, children with an *FLT3*-ITD AR > 0.1 may benefit from FLT3 inhibitor therapy and SCT in first remission with this lower threshold being used in upcoming trials.

**Table 1:** Recurrent mutations in pediatric AML. Duration of survival endpoint varies depending on study

Category	Mutation	Reference	Prevalence	Estimated EFS	Estimated OS	Special considerations
Tyrosine kinase	FLT3/ITD AR > 0.1	13, 20	15%	25–35%	60%	Co-occur with *WT1*, *DEK-NUP214*, and *NUP98* fusions
	FLT3-TKD	13	8–10%			
	RAS	13, 21	35–40%	65%	81%	*NRAS* mutations more common than *KRAS*
	KIT	13, 21	12%	60%	90%	Enriched in inv(16) and t(8;21) AML
Epigenetic modifiers	KMT2A fusions	9, 27	15–20%	44%	56%	Prognosis dependent on fusion partner
	t(9;11)		39–43%^a^	50%	63%	
	t(4;11)		1–2%^a^	29%	27%	
	t(6;11)		5–8%^a^	11%	22%	
	t(10;11)(p11.2q23)		1–2%^a^	17%	27%	
	t(10;11)(p12q23)		13%^a^	31%	45%	*MLL-MLLT10* fusion
	t(11;19)		12–14%^a^	46-49%	47-61%	
	MLLT10 fusion (non-KMT2A)	29, 30	< 1%		36%	EMD common
	DEK-NUP214	13, 17, 32	1–2%	32% 68%^b^	53%	High rates of induction failure More common in > 10 years of age
	KAT6A fusion	35	< 0.5%	57%	59%	Common in c-AML and may have spontaneous remission 66% with EMD
Transcription factors	WT1 mutation	10, 13	10–15%	30%	45%	Often co-occurs with *NUP98* fusion or *FLT3*/ITD
	ETV6 fusions	10, 30, 37, 44	< 1%	43%^b^	12% 100%^b^	Most common in < 18 months of age *MNX1-ETV6* found in 30% infant AML
	CBFA2T3-GLIS2	17, 41	2%	16.7%	41.7%	27% of M7 AML
	NUP98 fusion	13, 17, 21, 41	5–10%	13–17%	33.3–52%	9% of M7 AML
Others	FUS-ERG	17, 30	< 1%	9%	31%	
	MECOM fusion	30	< 1%	9%	31%	
	NPM1-MLF1	30	< 1%			

*EFS* event-free survival, *OS* overall survival, *AR* allelic ratio, *EMD* extra-medullary disease, *c-AML* congenital AML, *SCT* stem cell transplant

^a^Percentage of KMT2A rearrangements

^b^With SCT

#### RAS

Mutations in the Ras pathway are the most common mutations occurring up to 40% of pediatric AML patients, primarily in either *NRAS* or *KRAS* genes [13•]. Similar to *FLT3*, *RAS* mutations lead to constitutive activation of the Ras pathway. Despite intense research in associating *RAS* mutations with outcome, they do not independently alter relapse risk or survival measures in pediatric AML in general, but they may play a prognostic role in specific subtypes [21]. For example, in CBF AML, *RAS*-mutated patients have a lower risk of relapse, though this is not integrated into current risk stratification algorithms [22]. Importantly, *RAS* mutations may play a role as a therapeutic target as many new therapies utilize tyrosine kinase (TK) inhibition to limit cell growth. Mutations in Ras pathway genes are also found to associate with rearrangements in *KMT2A*, though this relationship appears to be most common in infant acute lymphoblastic leukemia [23].

#### KIT

Mutations in the tyrosine kinase receptor *KIT* gene occur in 12% of pediatric AML patients and typically lead to unregulated function of the KIT protein [10••, 13•, 24]. These mutations occur in two hotspot locations in the gene, exon 8 and exon 17, though only exon 17 mutations have definitively been shown to cause KIT auto-phosphorylation and constitutive activation [25]. Though previous studies have not determined a prognostic value of *KIT* mutations in pediatric AML as a whole, others have shown an improved OS associated with *KIT* mutations [13•, 21]. These recent results may be due to an enrichment of *KIT* mutations in the favorable-risk CBF AML group, as *KIT* mutations occur in 24–36% of this population with a slight predominance for exon 17 mutations [22, 26]. While studies in CBF AML as a whole also do not demonstrate a prognostic significance of *KIT* mutations, such mutations within the t(8;21) subgroup of AML increase the risk of relapse and lead to an inferior OS of only 50% at 4 years [22, 26].

### Epigenetic Modifiers

#### KMT2A Rearrangements

Lysine methyltransferase 2a encoded by *KMT2A* (formerly *MLL*) regulates gene expression via methylation of histone residues and epigenetic modifications. It is located on chromosome 11q23 and is frequently involved in gene rearrangements in AML with a multitude of fusion partners. *KMT2A* rearrangements were included in the original WHO AML classification, but the prognostic significance in adults was unclear. *KMT2A* rearrangements are more common in children than adults with a particularly high occurrence in infant AML, and the fusion partner profile is also unique to each age group [16, 27••]. While studies in children show that *KMT2A* rearrangements are clearly associated with inferior outcomes with an EFS of 44% and an OS of 56% along with higher rates of early death and relapse, the true prognostic value is highly dependent on the fusion partner gene (Table 1) [9, 15, 28]. The *KMT2A-MLLT3* fusion resulting from t(9;11)(p22;q23) is the most common *KMT2A* rearrangement in children, yet over 100 different fusion partners have been identified [15, 16]. Of the recurrent fusions, t(6;11)(q27;q23) and t(10;11)(p12;q23) have the worst outcomes with a 5-year OS of approximately 30% [9, 28]. Interestingly, *MLLT10*, the fusion partner in t(10;11)(p12;q23), also pairs with other genes in novel fusion events [10••, 29, 30•]. Other *KMT2A* rearrangements such as t(4;11)(q21;q23) and t(11;19)(q23;p13.3) are now considered HR fusions due to their associated high relapse risk [9, 28]. In contrast, one study demonstrated a 100% 5-year OS and a 92% EFS in 25 children harboring t(1;11)(q21;q23) [9]. Due to the low frequency of novel *KMT2A* fusions, not all rearrangements have well-defined outcomes, but careful consideration should be given to the fusion partner when considering the treatment approach for such patients.

#### DEK-NUP214 Fusion

AML with t(6;9)(p22;q34), a translocation leading to *DEK*-*NUP214* fusion, represents less than 2% of children with AML [31–33]. DEK is involved in chromatin structure and transcriptional regulation, whereas *NUP214* regulates transport of structures between the nucleus and cytoplasm [33]. In children, international studies demonstrate a high rate of induction failure with only 67% CR along with 5-year OS of 39–53%, EFS of 32%, and relapse rate of 57–64% [31, 32]. This fusion is more common in older children with a median age of 10.4 years. While some studies suggest that the presence *FLT3*-ITD does not independently alter outcomes in this subgroup, a recent Children’s Oncology Report (COG) report showed a superior EFS in those with t(6;9) that also harbored *FLT3*-ITD, likely due to the fact that all *FLT3*-ITD-positive patients received SCT in 1st CR [31, 32]. In both the American and European experiences, SCT in 1st CR for patients with t(6;9) significantly improved EFS to 68% compared to 0–18% in those who received chemotherapy alone, demonstrating a clear role for SCT in the treatment of *DEK-NUP214* AML [31, 32].

#### KAT6A Fusion

Translocations involving 8p11 are a newly described entity in AML, which lead to fusion of *KAT6A*, a histone acetyltransferase, with a partner gene, most commonly *CREBBP* on 16p13. In adults, this fusion is often found in younger patients with unique clinical characteristics such as leukemia cutis, disseminated intravascular coagulation, and hemophagocytosis [34]. In children, this rare fusion is strongly associated with congenital AML, presenting this way in over 25% of described cases [35]. Amongst all presentations of *KAT6A* fusions, current reports demonstrate a 5-year EFS and an OS of 55–60% in those who undergo therapy with curative intent and often undergo SCT in first remission [35]. Relapses tend to occur within the first year of diagnosis. Of particular interest is the natural progression in congenital AML with *KAT6A* fusions, where spontaneous remission often occurs, though 50% will subsequently have return of their disease [35].

### Transcription Factors and Other Fusions

#### WT1

Wilms’ tumor 1 (*WT1*) gene is commonly mutated across different malignancies and regulates quiescence in hematopoietic progenitors as well as differentiation of myeloid cells [21]. Mutations in *WT1* occur in approximately 10–15% of childhood AML and are more likely to be clonal than in adults [10••, 13•, 36]. In half of children with *WT1* mutations, both alleles are affected either by mutation or by deletion [36]. In addition, *WT1* mutations frequently co-occur with aberrations such as *FLT3*-ITD and *NUP98* fusions [21, 36]. *WT1* mutations are independent poor prognostic factors with a 5-year OS of 35% and EFS of 22% in children [36]. In addition, CR rates are significantly lower in children with *WT1* mutations [13•]. The combination of *WT1* mutation and *FLT3*-ITD confers an even poorer prognosis with an OS of 21% and EFS of 15–30% and a significantly higher rate of relapse, though these outcomes do not reflect the most recent clinical trials [10••, 21].

#### ETV6 Abnormalities

Fusions with *ETV6*, common in lymphoid leukemias, have also been identified in pediatric myeloid malignancies at a lower frequency. The fusion of *ETV6* with *MNX1*, resulting from t(7;12)(q36;p13), was originally described in infant AML and associated with an extremely poor prognosis with a 3-year EFS and OS of 0% [37, 38]. Recently, *MNX1*-*ETV6* has been identified outside of infancy where it frequently co-occurs with trisomy 19 and has better outcomes than previously reported [38]. While high rates of relapse have been consistently demonstrated, the 3-year OS in a recent European study was 100% as patients had improved outcomes with SCT following relapse [37]. *ETV6* has also been found to fuse with numerous other genes in pediatric AML and to be associated with inferior survival, leading to the recent inclusion of *ETV6* fusions in the HR genetic stratification [10••, 30•]. In addition to fusions, loss of *ETV6* via chromosome 12p deletion is also associated with a poor prognosis [16].

#### CBFA2T3-GLIS2

*CBFA2T3*-*GLIS2* fusion results from a cryptic translocation in chromosome 16 [39]. It was first described in children with non-Down syndrome acute megakaryoblastic leukemia (AMKL) where it occurs in 30% of children, but has recently been described in approximately 8% of AML patients less than 3 years of age [39–41]. *CBFA2T3-GLIS2* has a predisposition for African American children which make up nearly 1/3 of patients with the fusion [40]. These children have previously been treated with standard therapy as the *CBFA2T3-GLIS2* fusion does not co-occur with traditional HR karyotypes [40]. On retrospective analysis of clinical trials, children with *CBFA2T3-GLIS2* had far inferior outcomes with only 50% achieving CR at the end of first induction and 35% having persistent MRD after induction 2. Both EFS and OS are poor at only 20–40% at 5 years regardless of having the AMKL phenotype [17, 39]. These children reflect a previously undefined group of HR children who may benefit from early SCT.

#### NUP98 Fusions

*NUP98*, a nucleoporin gene, has been of recent interest due to its fusion with *NSD1* in t(5;11)(q35;p15) in less than 5% of pediatric AML patients [13•, 21]. *NUP98-NSD1* is not found in combination with other cytogenetic rearrangements, but it frequently co-occurs with *FLT3*-ITD or *WT1* mutations, with one or both genes mutated in 80% of patients [13•, 21, 24, 42]. *NUP98* fusions in children independently increase the risk of relapse and decrease CR, EFS, and OS [13•, 21, 42]. *NUP98* fusions combined with either *FLT3*-ITD or *WT1* mutations have an even worse prognosis than *NUP98* fusions alone, with a relapse rate over 75% and an OS of 20–40% [21, 42]. Clinically, these fusions are associated with a higher presenting white blood cell (WBC) count and older patient age [43]. A second recurrent fusion with *NUP98* is the *NUP98-KDM5A* fusion, identified in 2% of children from recent American and European trials [43]. In contrast to *NUP98-NSD1*, the *NUP98-KDM5A* fusion is more common in young children and most common in AMKL where it was originally described [17, 43]. The *NUP98-KDM5A* fusion rarely co-occurs with *WT1* or TK mutations, yet still has a very poor prognosis. The 5-year EFS and OS of this group are 16–39% and 33–34%, respectively [17, 43]. Importantly, of those who underwent SCT in 1st remission, 71% still subsequently relapsed, demonstrating an ongoing need for better therapies [17]**.**

#### Other Mutations

Additional rare gene fusions and mutations exist which are associated with inferior survival. However, the small number of children in which these mutations occur has limited the ability to study such genetics in detail, including accurate survival statistics [30•, 44]. These include *RPN1*- and *RUNX1-MECOM* fusions, *NPM1-MLF1*, and *FUS-ERG* [17, 30•]. Many of these fusions occur due to cryptic rearrangements and were previously unidentified when use of fluorescent in situ hybridization was the predominant means of diagnosing genetic abnormalities. The use of advanced genetic testing strategies, such as RT-PCR, has since improved the detection of these negative prognostic markers and is an essential tool to correctly assess patient risk [10••, 30•, 44]. Recognition of each of these HR genetic features in pediatric AML is necessary to improve outcomes.

## New Therapy

### Current Treatment Approach

While outcomes in pediatric AML have improved over the decades, these advances have been primarily due to early intensification of therapy, early use of SCT in HR patients, and improvements in supportive care. Current regimens still rely heavily on high-dose chemotherapy including cytarabine and anthracyclines in order to induce remissions. These agents carry a serious risk of toxicity including infection and cardiac dysfunction. Despite high doses of cytotoxic chemotherapy, relapse remains a frequent problem in pediatric AML. The newer treatment approaches discussed below have only recently been introduced in pediatric clinical trials (Table 2). These agents were typically developed and studied in older adults unable to tolerate the intensity of standard therapies, including SCT, and are now being translated into pediatric AML care.

**Table 2:** New therapeutics in pediatric AML

Drug	Mechanism of action	Current stage of development in pediatric AML
Drug-antibody conjugates		
Gemtuzumab ozogamicin [14]	Anti-CD33 antibody with calicheamicin payload	FDA-approved for newly diagnosed AML > 1 month of age Standard of care as single dose in induction 1
Flotetuzumab [58]	CD123/CD3 bispecific dual-affinity retargeting antibody	Phase I trials for r/r AML
Nivolumab [61]	Anti-PD-1 antibody/checkpoint inhibitor	Phase I/II trial for r/r AML combined with azacitidine
Epigenetic modifiers		
Decitabine [67]	Hypomethylating agent: inhibits DNA methyltransferases	Completed phase I trial for r/r AML Phase II trial with standard chemotherapy in newly diagnosed AML
Azacitidine [68]	Hypomethylating agent: inhibits DNA methyltransferases	Phase I/II trials for r/r AML Phase II trial with standard chemotherapy in newly diagnosed AML
Vorinostat [72]	Inhibits histone deacetylase	Phase I trial for r/r AML
Panobinostat [71]	Inhibits histone deacetylase	Phase I trial for r/r AML
Tyrosine kinase/*FLT3* inhibitors		
Sorafenib [80]	1st-generation type II TKI: active against *FLT3-*ITD and *FLT3*-TKD mutations	Completed phase III trials for high AR *FLT3-*ITD AML
Midostaurin [83]	1st-generation type I TKI: active against *FLT3* and *KIT* mutations	USA: phase I/II trials terminated for low enrollment International: ongoing phase II trial
Gilteritinib [87]	2nd-generation type I TKI: active against *FLT3* and *AXL*	Phase III trials combined with standard chemo in newly diagnosed AML Phase I/II trial combined with FLAG for r/r AML
Quizartinib [75]	Type II TKI	Phase I/II trials in r/r AML
Crenolanib [75]	Type I TKI	Completed phase I trial in r/r leukemias
Others		
Venetoclax [99]	Inhibits BCL-2	Phase I/II trials for r/r AML
CPX-351 [99]	Liposomal formulation of daunorubicin and cytarabine—enhances synergy between drugs and extends half-life	Completed phase I/II trials for r/r AML Current phase III trial for newly diagnosed AML
Atovaquone [99]	Inhibits oxidative phosphorylation and STAT3 activation	Phase I trial with standard chemotherapy
Chimeric antigen receptor T cells		
CD123-targeting CAR-T [62]	T cells genetically modified to target/kill CD123-expressing AML cells	Phase I trials for r/r AML
CD33-targeting CAR-T [46]	T cells genetically modified to target/kill CD33-expressing AML cells	Phase I trials for r/r AML

*FDA*, Food and Drug Administration; *r/r*, relapsed/refractory; *TKI*, tyrosine kinase inhibitor; *ITD*, internal tandem duplication; *TKD*, tyrosine kinase domain; *AR*, allelic ratio; *FLAG*, fludarabine, cytarabine, granulocyte colony-stimulating factor; *CAR-T*, chimeric antigen receptor T cell

### Immune-Based Therapy

#### Drug-Antibody Conjugates

Targeted therapy which exploits cell surface markers unique to cancer cells has been a significant therapeutic advance in cancer treatment. In drug-antibody conjugates, antibody targeting can deliver a drug, such as the small molecule calicheamicin, directly to cells of interest. Gemtuzumab ozogamicin (GO) is one such treatment where calicheamicin is conjugated to an anti-CD33 antibody and has potent anti-tumor effects on CD33-expressing cells, found in a majority of AML cases [45, 46]. Despite initial adult studies demonstrating improvements in EFS and OS when used in upfront or relapse therapy, GO was pulled from the market due to its increased toxicities [47, 48]. More recent studies have demonstrated that lower doses of GO can produce similar outcomes with less treatment-related mortality, leading to re-approval of GO in adult AML therapy [49, 50]. GO was subsequently incorporated into pediatric clinical trials in both relapsed and upfront therapies where it improved EFS and reduced relapse risk; however, OS and CR rates remained unchanged [14, 51]. The reduced relapse risk (32.8% vs. 41.3%) when GO was given in upfront therapy in COG AAML0531 was offset by increased treatment-related mortality (8.6% vs. 5.9%) [14]. Newer studies are therefore designed to maximize benefit while limiting toxicity by giving GO as a single dose in induction, as this has been shown to have the highest benefit and may reduce toxicity profiles [14, 52]. Children more likely to benefit from GO therapy include those with *FLT3*-ITD mutations, *KMT2A* rearrangements, single-nucleotide polymorphisms in *ABCB1* and *CD33*, and high CD33 expression, but these are not currently factored into treatment decisions [18, 53–55].

CD123, the IL-3 receptor α-chain, has also been a cell surface target of interest in AML owing to its expression in a majority of AML cases, including leukemic stem cells [56]. CD123-targeted therapies remain in the early phases of development but may provide an additional avenue of treatment. To date, several antibody conjugates have been explored, including a CD123-targeting drug conjugate in which an anti-CD123 antibody is linked to the alkylating agent indolinobenzodiazepine pseudodimer and a dual-affinity retargeting (DART) CD3-CD123 platform which assists CD3^+^ T cells in recognizing and eliminating CD123-expressing cells [57, 58]. The CD3-CD123 DART is currently in pediatric early-phase clinical trials.

Interest has also recently grown in the use of checkpoint inhibitors in AML to enhance the immune response to cancerous cells. This strategy has been used successfully in adult solid tumors but remains experimental in AML. Currently available checkpoint inhibitors include nivolumab and pembrolizumab, anti-PD-1 antibodies, and ipilimumab, which targets CTLA-4. Early clinical trials in adult AML have shown that checkpoint inhibition, either as monotherapy or in combination with other treatments, is a feasible and safe strategy now being explored in larger studies [59]. Nivolumab has also been successfully used in adults who relapse following SCT to regain donor chimerism without the need for second transplant [60]. The only reported use in pediatric AML was in a child with highly refractory relapsed AML in combination with azacitidine in which the child had symptomatic relief after therapy but failed to have any significant improvement in disease burden, though phase I/II clinical trials in children with AML have recently begun (NCT03825367) [61].

#### Chimeric Antigen Receptor T Cells

Immunotherapy with chimeric antigen receptor T cells (CAR-T) for AML has lagged significantly behind lymphoid leukemias in development and effectiveness. The heterogeneity of AML has made it challenging to identify adequate leukemia-specific antigens which do not also produce on-target off-tumor effects on normal tissue. CD33 and CD123 are expressed in 90% and 75% of AML, respectively, with less than 5% of cases being negative for both [56]. While CD33-directed therapies also target non-leukemic mature and progenitor myeloid cells, the lack of CD33 expression in hematopoietic stem cells coupled with the success of GO has provided rationale for investigation into the use of CD33-directed CAR-Ts [46]. CD123 is expressed at high levels in multiple types of hematologic malignancies, making it an attractive target across leukemia types [62]. However, CD123 is also expressed on normal hematopoietic stem cells and may therefore be most useful as a bridge to SCT. Both CD33- and CD123-directed CAR-T trials are currently underway in pediatric studies for relapsed/refractory AML (NCT04318678, NCT03971799). Due to concerns for on-target off-tumor effects, it is likely that sophisticated targeting strategies such as dual-targeting or switchable CAR-Ts will be required to avoid serious toxicity [56, 62].

### Epigenetic Modifiers

#### Hypomethylating Agents

The prevalence of epigenetic dysregulation in AML development has led to a rising interest in targeting epigenetic modifiers as part of therapy. Hypomethylating agents (HMAs) such as decitabine and azacitidine are DNA methyltransferase (DNMT) inhibitors which alter DNA methylation patterns, leading to increased expression of tumor suppressors and apoptosis [63]. In adults, these therapies prolong survival but rarely lead to sustained remission when used as a single agent [64]. Decitabine has also been used as maintenance therapy for adults with AML but did not protect against relapse when used in this setting [65]. In children, case reports and small early-phase trials incorporating DNMT inhibitors have demonstrated reasonable safety profiles and efficacy in the relapse setting, but large studies have not been completed [66, 67]. One study utilized azacitidine in combination with fludarabine and cytarabine chemotherapy and achieved CR in 7/12 children with relapsed or refractory AML [68]. The use of HMAs in upfront treatment with cytotoxic chemotherapy is currently being investigated in the St. Jude AML16 trial (NCT03164057).

#### HDAC Inhibitors

Histone deacetylases (HDACs) contribute to epigenetic regulation via the removal of acetyl groups from histones condensing chromatin and decreasing gene transcription. Inhibition of HDAC activity remodels chromatin in AML cells with attendant decreased expression of DNA repair genes, depletion of CXCR4, cell cycle arrest, and ultimately apoptosis, with these effects being synergistic with standard chemotherapy regimens [69, 70]. The two HDAC inhibitors which have been investigated in AML include vorinostat and panobinostat which are FDA-approved for refractory multiple myeloma and cutaneous T cell lymphoma, respectively. Preclinical studies demonstrate that the effects of HDAC inhibitors are maximized when given with other drugs such as HMAs where they serve as epigenetic sensitizers [71, 72]. While adult studies have had mixed results, HDAC inhibitors can be safely combined with other agents such as DNMT inhibitors and relapse regimens and are currently being investigated in relapsed pediatric AML (NCT03263936) [73, 74].

### FLT3 Inhibitors

*FLT3*-mutated AML has been a specific disease subset of interest due to the targetable nature of tyrosine kinases. FLT3 inhibitors are small molecules which bind to the adenosine triphosphate–binding site on FLT3 leading to competitive inhibition of kinase activity [75]. The drugs are categorized as type I or type II inhibitors with both affecting ITD mutations, but type I inhibitors are also active against TKD mutations and have higher binding affinity for FLT3 [76]. In addition, first-generation inhibitors have multikinase activity with lower specificity for FLT3 and more off-target effects, whereas second-generation inhibitors are more specific for FLT3 activity. There are at least 8 FLT3 inhibitors currently on the market or under development, though none is approved for use in children. Sorafenib is a multikinase type II inhibitor and one of the first used in the treatment of AML. When combined with chemotherapy, sorafenib improved CR and EFS regardless of *FLT3* mutation status in younger adults with AML, but also increased toxicity [77, 78]. Early-phase clinical trials in children with relapsed AML demonstrated that sorafenib was tolerable and effective when given with chemotherapy [79]. Sorafenib was therefore added to treatment of children with newly diagnosed, high AR *FLT3*-ITD AML, including 1 year of maintenance therapy in the most recent COG trial. Preliminary results demonstrate that addition of sorafenib increases CR and EFS and decreases relapse risk in children with high AR *FLT3*-ITD, providing rationale for ongoing use of FLT3 inhibitors in children [80].

Midostaurin, the first FDA-approved FLT3 inhibitor, is a first-generation type I FLT3 inhibitor with off-target effects on KIT and was shown to decrease blast percentages in patients with relapsed/refractory AML when used as a single agent regardless of *FLT3* mutation status [81]. The large multinational RATIFY study demonstrated that standard chemotherapy (plus SCT) plus midostaurin prolonged survival and decreased relapse risk compared to chemotherapy and SCT alone [82]. Preclinical studies of midostaurin in patient-derived xenograft models were promising, but clinical trial use has been limited by low enrollment and the introduction of second-generation inhibitors [83].

Gilteritinib is a second-generation type I FLT3 inhibitor which also inhibits AXL, a tyrosine kinase which enhances FLT3 activation and serves as a mechanism for FLT3 inhibitor resistance [84]. It was FDA-approved in adults with relapsed/refractory *FLT3*-mutated AML after demonstrating a tolerable safety profile with a 20–30% CR rate and significantly prolonged survival when given as monotherapy [85, 86]. Preclinical models showed that the combination of gilteritinib with either azacitidine or cytarabine and anthracyclines further potentiated anti-leukemic effects [87]. While pediatric studies are currently lacking, two upcoming pediatric trials will investigate the use of gilteritinib in *FLT3*-mutated AML in combination with chemotherapy in both the relapse and upfront settings (NCT04240002, NCT04293562). Newer FLT3 inhibitors such as quizartinib and crenolanib are currently being investigated as well and appear to have similar efficacy to the approved FLT3 inhibitors [88].

### Other Therapeutics

#### BCL-2 Inhibition

Venetoclax inhibits BCL-2, an anti-apoptotic protein whose overexpression is a mechanism of resistance in AML [89]. In adults, venetoclax with HMAs or low-dose cytarabine is both tolerable and improves response rates when used as part of low-intensity front-line therapies, but its utility in relapse remains unclear [90]. Pediatric studies are still in early phases, but initial studies demonstrated a CR of 70% when used in combination with cytarabine with or without idarubicin [91]. Preclinical studies also suggest that venetoclax works synergistically with FLT3 inhibitors midostaurin and gilteritinib to induce apoptosis in AML cells, and may provide another therapeutic avenue [92].

#### Liposomal Chemotherapy

A liposomal formulation of traditional chemotherapy agents cytarabine and daunorubicin, termed CPX-351, optimizes pharmacodynamics and synergistic effects via maintenance of the optimal 5:1 molar ratio which extends the half-life and enhances uptake in the bone marrow [93, 94]. The use of CPX-351 in adults demonstrated superior remission rates compared to conventional chemotherapy, though improvements in EFS and OS were limited to those with secondary AML [95, 96]. In children, a phase I/II study in relapsed/refractory AML demonstrated a high response rate with 75% achieving a CR after a single course of CPX-351, 80% of whom were negative for MRD [97•]. A total of 96.7% of patients with a CR subsequently received additional relapse therapy with fludarabine, cytarabine, and granulocyte-colony-stimulating factor followed by SCT. The OS for the entire patient population was 52.7% at 2 years from study entry. While CPX-351 has demonstrated promising early results and is being incorporated into upfront therapy for HR children in upcoming trials, it maintains a similar side effect profile to the conventional formulations of daunorubicin and cytarabine [95, 96, 97•].

#### Oxidative Phosphorylation Inhibitors

Other therapeutic strategies rely on rebranding older medications for new purposes, such as atovaquone, an antibacterial and antimalarial medication which inhibits electron transport and is approved for use in the prevention of *Pneumocystis jirovecii* pneumonia (PJP) [98]. Recent studies have shown that atovaquone has effects on AML cells by inhibiting STAT3 phosphorylation and subsequent expression of STAT3 target genes [99]. In preclinical studies, atovaquone induces apoptosis in AML cells, inhibits the mechanistic target of rapamycin, and inhibits oxidative phosphorylation (oxphos), leading to prolonged survival in patient-derived xenograft models [100]. In adults, atovaquone is commonly used in post-SCT for PJP prophylaxis, and one retrospective study demonstrated that adults with AML who underwent SCT with prolonged atovaquone use had lower relapse rates than those with shorter atovaquone exposure, providing further rationale for clinical use of atovaquone for AML [99]. In children, an ongoing multi-institution feasibility study uses atovaquone for PJP prophylaxis in AML patients receiving traditional chemotherapy (NCT03568994). Metformin and lonidamine have also been identified as oxphos inhibitors with anti-tumor effects in vitro, though lonidamine is not commercially approved in the USA [101].

## Conclusion

The outcomes in pediatric AML, while improved in recent decades, remain suboptimal with high rates of relapse and few options for therapy when initial treatment regimens fail. Genetic risk grouping has gone a long way to target the intensity of therapy to those at the highest risk of relapse and death; however, the heterogeneous nature of AML has left many HR genetic features unidentified until recently.

Through the use of comprehensive mutation testing, collaborative international efforts, and integration of data from adult trials, critical information is now known regarding even rare genetic lesions that alter outcomes. This has primarily led to a growing list of HR genetic features. Specific *KMT2A* fusions, *DEK-NUP214* and *WT1* mutations, *ETV6* rearrangements, and *CBFA2T3-GLIS2*, *MECOM*, *MLLT10*, and *NUP98* fusions are now included in various cooperative group risk algorithms that recommend SCT in first CR. The potential for benefit of SCT transplant in first CR for these patients remains to be seen. Additionally, challenges surrounding attainment of an MRD-negative CR using traditional chemotherapy may limit the utility of this approach and continue to highlight the need for additional treatment options for such patients.

The treatment of AML is quickly changing in adults, yet the advances in pediatrics are slower to evolve due to a lack of drugs developed specifically for the pediatric population and the challenges of evaluating novel agents for a relatively rare disease. Standard therapies in adult AML, such as GO and FLT3 inhibitors, have only recently been incorporated into pediatric trials, and the optimal way in which to incorporate them into therapy is still under investigation. In addition, newer agents such as HMAs and DNMT inhibitors have been developed primarily for adults who cannot tolerate intensive cytotoxic therapy, and therefore, acceptable outcomes in this population differ greatly from the desired results in children. However, the use of these agents may prove beneficial when incorporated into relapse regimens in children with pediatric AML.

The continual expansion of recurrent genetic lesions in AML and their effects on outcomes, along with the growing list of therapeutic options, may ultimately provide an opportunity to individualize regimens in pediatric AML. The addition of GO and FLT3 inhibitors to standard chemotherapy regimens is now becoming a standard of care, but more studies are needed on other new agents to determine their utility in children. Through ongoing collaboration and innovation, treatment strategies will become more tailored to the underlying genetics of the disease with resultant improvements in outcome.
